# Acceleration of Magnetic Resonance Fingerprinting Reconstruction Using Denoising and Self-Attention Pyramidal Convolutional Neural Network

**DOI:** 10.3390/s22031260

**Published:** 2022-02-07

**Authors:** Jia-Sheng Hong, Ingo Hermann, Frank Gerrit Zöllner, Lothar R. Schad, Shuu-Jiun Wang, Wei-Kai Lee, Yung-Lin Chen, Yu Chang, Yu-Te Wu

**Affiliations:** 1 Department of Biomedical Imaging and Radiological Sciences, National Yang Ming Chiao Tung University, Taipei 112, Taiwan; eternityjh.be06@nycu.edu.tw (J.-S.H.); l850818.be07@nycu.edu.tw (W.-K.L.); 2 Computer Assisted Clinical Medicine, Mannheim Institute for Intelligent Systems in Medicine, Medical Faculty Mannheim, Heidelberg University, 68167 Mannheim, Germany; Ingo.Hermann@medma.uni-heidelberg.de (I.H.); frank.zoellner@medma.uni-heidelberg.de (F.G.Z.); Lothar.Schad@medma.uni-heidelberg.de (L.R.S.); 3 Department of Neurology, Neurological Institute, Taipei Veterans General Hospital, Taipei 112, Taiwan; sjwang@vghtpe.gov.tw; 4 College of Medicine, National Yang Ming Chiao Tung University, Taipei 112, Taiwan; 5 Brain Research Center, National Yang Ming Chiao Tung University, Taipei 112, Taiwan; 6 Institute of Biophotonics, National Yang Ming Chiao Tung University, Taipei 112, Taiwan; thomaschen83.be08@nycu.edu.tw (Y.-L.C.); changyu97@gm.ym.edu.tw (Y.C.)

**Keywords:** magnetic resonance fingerprinting, echo-planar imaging, T1 and T2* relaxation times, denoising convolutional neural network, self-attention, feature pyramid network

## Abstract

Magnetic resonance fingerprinting (MRF) based on echo-planar imaging (EPI) enables whole-brain imaging to rapidly obtain T1 and T2* relaxation time maps. Reconstructing parametric maps from the MRF scanned baselines by the inner-product method is computationally expensive. We aimed to accelerate the reconstruction of parametric maps for MRF-EPI by using a deep learning model. The proposed approach uses a two-stage model that first eliminates noise and then regresses the parametric maps. Parametric maps obtained by dictionary matching were used as a reference and compared with the prediction results of the two-stage model. MRF-EPI scans were collected from 32 subjects. The signal-to-noise ratio increased significantly after the noise removal by the denoising model. For prediction with scans in the testing dataset, the mean absolute percentage errors between the standard and the final two-stage model were 3.1%, 3.2%, and 1.9% for T1, and 2.6%, 2.3%, and 2.8% for T2* in gray matter, white matter, and lesion locations, respectively. Our proposed two-stage deep learning model can effectively remove noise and accurately reconstruct MRF-EPI parametric maps, increasing the speed of reconstruction and reducing the storage space required by dictionaries.

## 1. Introduction

Quantitative magnetic resonance (MR) relaxometry can quantify the relaxation time (e.g., T1, T2, T2* relaxation time) to clarify the physical and pathological properties of human tissues [1]. Quantitative MR relaxometry was reported to increase accuracy and precision compared with conventional weighted magnetic resonance imaging (MRI) in detecting lesions, and it can even synthesize traditional weighted images [2,3]. However, clinical applications of quantitative MR relaxometry are limited by the length of the imaging procedure required to estimate the tissue relaxation time; moreover, motion artifacts can interfere with the results, and the procedure does not meet the needs for clinical scheduling efficiency. Magnetic resonance fingerprinting (MRF) is an approach for designing the rapid quantitative sequence [4]. MRF has the advantage of providing quantitative images of multiple types of relaxation times simultaneously in a relatively short imaging time (several minutes). However, because MRF image reconstruction requires comparison with a vast computer simulation database (dictionary matching), the extended image reconstruction time has become a considerable challenge in the development of MRF [5].

The dictionary matching process is computationally expensive and requires storage space for the simulation database, which hinders clinical applications of MRF. Thus, optimizing the MRF signal matching process is crucial. Toward this aim, dimension reduction algorithms, such as singular value decomposition, were the first to be used. Studies have used singular value decomposition to project the database into low-dimensional space, speeding up the MRF signal matching process by 3.4–4.8 times that of using only the inner-product method [6,7]. Compared with approaches reducing the dimensionality of the database, a model trained by deep learning can eliminate the storage usage of the MRF simulation database and achieve near real-time reconstructions. Recent studies on the use of deep learning to accelerate the MRF reconstruction process have included the use of a one-dimensional (1D) neural network, a convolutional neural network (CNN), and a recurrent neural network (RNN) to train models for learning the simulated information [8,9,10]. Studies have also modeled the reconstructed images in a two-dimensional (2D) fashion by using the data after matching the dictionary with the scanned images [11,12,13]. Moreover, deep learning models can combine multiple tasks, including the reconstruction of MRF parametric maps, preprocessing, and tissue segmentation, thus reducing computation times from hours to seconds [13]. Deep learning is therefore an efficient method for MRF image reconstruction. In addition to deep learning studies of MRF reconstruction, one study used generative adversarial networks to speed up the generation of simulation data [14]. As the graphics hardware and deep learning algorithms mature, MRI imaging techniques can be optimized with deep learning to improve computational performance and thus increase the feasibility of clinical applications [2].

Most deep learning studies for the MRF image reconstruction have developed their models based on the original MRF protocol by Ma et al., which has a signal with long time steps (a thousand-time points) [4]. Therefore, most models are designed to reduce the time dimension. For instance, Fang et al. used a two-stage deep learning strategy that entailed first extracting features through a fully connected neural network and then training the U-Net-based model to learn the spatial distribution of the brain tissue [12]. The feature extraction step is a process of reducing high-dimensional data to low-dimensional data. Longer time steps can compensate for the effects of noise, but for MRFs with shorter time steps, such as those used in this study (35-time points), the effects of noise cannot be underestimated. Cohen et al. demonstrated the extent to which noise affected the accuracy of their model, but they did not specifically design the model for noise reduction [8]. In addition, the selection of training and testing data is another critical point for training MRF models. Cohen et al. trained their model by simulation dictionary and tested using a digital brain phantom [8]. Hoppe et al. developed their CNN-based model by simulation dictionary and tested using a quantitative phantom. Chen et al. also devised a CNN-based model and tested their model by using the human scan data from another quantitative MRI method [15]. For the study using the same MRF protocol as this study, they only used scan data and did not include the simulation dictionary for training [13]. Their model performance had a between 5% and 10% error. Ideally, the deep learning model should be trained with the simulation dictionary, and the performance of the dictionary learning model is tested with human scan data. A dictionary learning model ensures that the model has learned all the possible situations, and models tested with human scan data are more convincing. Therefore, we designed and trained our model for noise reduction and used the dictionary learning model to predict human scan data to verify the performance of the proposed model.

MRF image reconstruction is a regression task for deep learning models, and the presence of noise affects the model performance [16]. A denoising CNN model (DnCNN) was proposed for image denoising; it is highly effective in general image denoising tasks [17]. Furthermore, the model can complete denoising tasks with an unknown noise level. Because dictionary matching is performed using the 1D approach, we modified the DnCNN for 1D signal denoising for the first stage of the proposed model. For the second stage of the model, which was aimed at learning the Bloch equation simulation [18], we designed a pyramidal model to extract features of the MRF signal evolution. A pyramid CNN exhibited promising performance in object detection tasks [19], and the advantage of the pyramid architecture is that it can extract and combine features from various scales. In addition, the self-attention mechanism has been used in natural language processing and can achieve state-of-the-art performance [20]. A CNN with self-attention can associate each pixel in a 2D image to generate a global reference between pixels [21]. We thus added the self-attention layer to the model for focusing on the connection between features extracted by the CNN. The weight of important features can be enhanced through the self-attention mechanism.

This study aimed to develop a deep learning model to replace the computationally expensive inner-product method for MRF reconstruction. We investigated how precisely the proposed model learned the Bloch equation simulation [18] and the relationship between the noise and model performance with scanned data. In the present study, MRF-echo-planar imaging (MRF-EPI) was used to scan the whole brains of 32 subjects to obtain T1 and T2* parametric maps [22,23,24]. Herein, we propose a two-stage model that first reduces MRF signal noise and then reconstructs parametric maps of MRF by a dictionary-learning model.

## 2. Materials and Methods

### 2.1. Population

The relevant institutional review board (2019-711N) approved this study, and the subjects provided informed consent before undergoing scanning. The MRF scan was implemented using a 3T scanner (Magnetom Skyra, Siemens Healthineers, Erlangen, Germany) with 14 healthy subjects and 18 subjects with multiple sclerosis (MS). The healthy group comprised eleven men and three women (aged 22–33 years; mean: 26 years). The MS group contained seven men and eleven women (aged 23–73 years; mean: 39 years). The scans of 32 subjects were used to evaluate the proposed model and are referred to as the “scanned data”.

### 2.2. Magnetic Resonance Fingerprinting Imaging and Dictionary Generation

The acquisition method used was a previously proposed and validated MRF-EPI imaging sequence [13,22,23,24]. The imaging parameters of the MRF sequence were as follows: in-plane spatial resolution = 1 × 1 mm^2^; slice thickness = 2 mm; bandwidth = 998 Hz/px; GRAPPA factor = 3; partial Fourier = 5/8, variable flip angle (34°–86°), echo time (21–81.5 milliseconds [ms]), repetition time (3530–6570 ms), and fat suppression. The acquisition time was 4 min 23 s for 60 slices of the whole brain. In addition, using the same spatial resolution, fluid-attenuated inversion recovery (FLAIR) was obtained for lesion segmentation. The MRF dictionaries were generated for each slice, with 598,842 entries based on the design of MRF-EPI using the Bloch equation simulation [18]. The ranges of T1 and T2* values were 100–4000 ms and 10–3000 ms (excluding those T1 smaller than T2*), respectively, with a 2% spacing. The range of flip angle efficiency B1+ was 0.6–1.4 with a 0.05 spacing.

**Figure 1:**
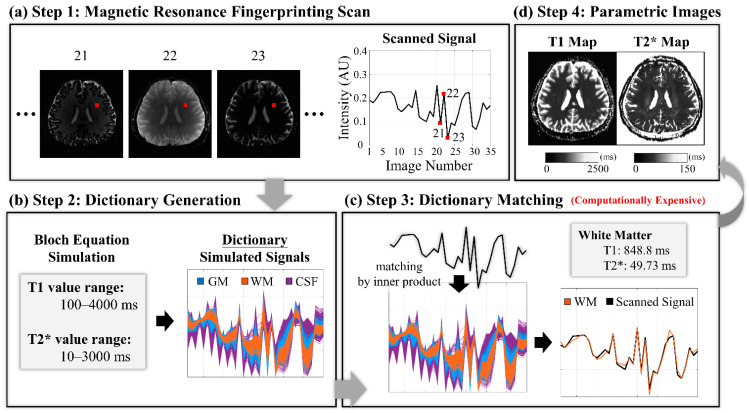
Schematic of the reconstruction for T1 and T2* maps of the magnetic resonance fingerprinting. (**a**) MRF baseline scan. (**b**) Dictionary generation process. (**c**) Dictionary matching by the inner product. (**d**) Parametric maps after matching pixel by pixel. MRF = magnetic resonance fingerprinting; AU = arbitrary unit; GM = gray matter; WM = white matter; CSF = cerebrospinal fluid.

The T1 and T2* maps of the scanned data were reconstructed by the inner-product method based on the 2%-increment dictionary. Figure 1 displays the schematic process of the MRF imaging. There were four steps in the MRF imaging process. The first was the MRF-EPI scan, which had a total of 35 images for each slice in which each pixel can be considered as a signal with 35 values (Figure 1a). Every pixel has its specific signal evolution that depends on the T1 and T2* relaxation times for the tissue of that pixel. The second was the dictionary generation, and the simulated dictionary was generated using the Bloch equation [18], given a certain range of T1 and T2* values (Figure 1b). The third was dictionary matching, where the MRF scanned signals were matched to the simulated dictionary signals one by one using the inner product (Figure 1c). When each pixel was matched, the parametric images were obtained, as in step 4 (Figure 1d). The time required for dictionary matching in the third step depends on the size of the dictionary in the second step. The denser the dictionary is, the more signal entries there are, and the longer the matching time is. This is where the challenge of MRF image reconstruction lies.

### 2.3. Dictionaries and Image Preprocessing

We separated the dictionaries with the 2% increment in the simulation into training and validation datasets using two divisions. The first division split the training and validation datasets by the T1 and T2* value range. T1 and T2* values were 500–2500 ms and 50–1500 ms, respectively, for the training, and the other entries were used for the validation. In this division, we aimed to test whether the deep learning model was able to learn Bloch equation simulation [18] to predict relaxation times that were not in the training range.

In the second division, the training and validation datasets were divided according to the incremental spacing of the T1 and T2* values (i.e., 4%, 6%, 8%, …, 20%). We sampled the entries by different intervals in the 2%-increment dictionary (i.e., 2, 3, 4, …, 10) to obtain dictionaries with the mentioned increment as a training dataset and the remaining unsampled ones as a validation dataset. For instance, the 2%-increment dictionary had T1 values of 100 ms, 102 ms, 104.04 ms, …, to the end, and T2* values of 10 ms, 10.2 ms, 10.404 ms, …, to the end. We sampled the T1 values of 100 ms, 104.04 ms, …, to the end, and then sampled the T2* values 10 ms, 10.404 ms, …, to the end, obtaining a 4%-increment dictionary for training. Other unsampled entries, T1 values 102 ms, …, to the end, and T2* values 10.2 ms, …, to the end, were used as validation data. In this division, we aimed to test how accurate the deep learning model was in predicting the relaxation times in the training range.

To compare the reconstructed result between the standard dictionary matching and the proposed model for different tissues, manual and automatic segmentation of different brain tissues was performed. Lesion locations for the MS group were manually segmented on FLAIR images by an expert radiologist. We used the SPM12 [25] to automatically segment the white matter (WM), gray matter (GM), and cerebrospinal fluid (CSF) from the T1 map obtained through MRF. A threshold of 80% of the maximum value was applied to the probability maps generated by SPM12 to create binary masks.

### 2.4. Noise Analysis and Denoising CNN

**Figure 2:**
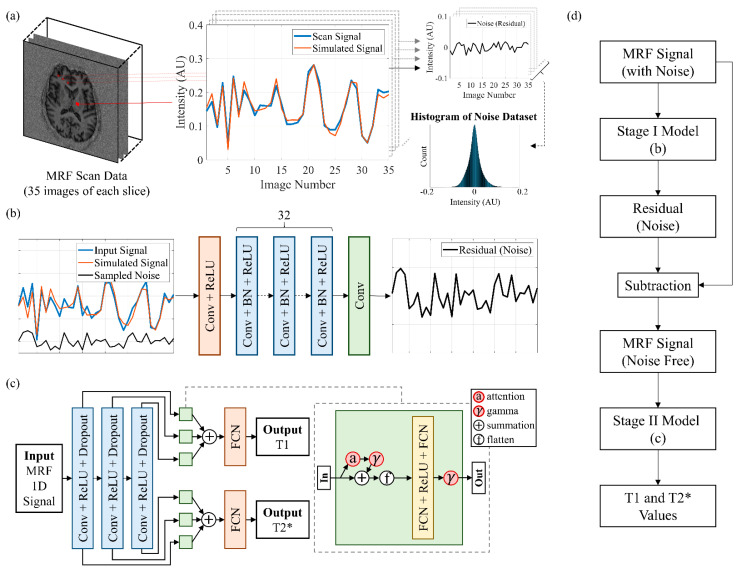
Schematic of the noise collection, denoising CNN, pyramid model, and flowchart of the two-stage model. (**a**) Collection of the noise dataset. AU = arbitrary unit. (**b**) Denoising CNN. (**c**) Weighted pyramid dual-path CNN with attention. (**d**) Flowchart of the successive process of the proposed model.

According to the inner product, the MRF scanned signals obtained from the subjects were first matched to the 2%-increment dictionary, which was the densest in our experiment. The matched signal from the simulated dictionary was considered as the noise-free signal. The signal without noise was subtracted from the scanned signal to obtain the residual for calculating the signal-to-noise ratio (SNR) as follows:

(1) $$\mathrm{SNR}=10\times \log_{10}\frac{\sum_{i=1}^{k}s_{i}^{2}}{\sum_{i=1}^{k}n_{i}^{2}},$$

where *s* and *n* are the matched signal from the simulated dictionary and the residual gathered by the difference between the scanned and matched signal, respectively; *k* is the length of the signal, which was 35 in our case. The SNR is in decibels (dB). We collected the amplitudes of residuals from 21 subjects (3 healthy subjects and 18 patients), slice by slice, and this collection was referred to as the “noise dataset” for training the denoising model. The temporal order of each residual was useless and thus discarded. The scans of the other 11 subjects were used as the testing dataset for evaluating the denoising model. Figure 2a displays a schematic of how the noise was obtained and collected.

Figure 2b displays the feedforward denoising CNN proposed for image denoising [17]. The denoising CNN was modified for noise reduction of 1D signals in this study. The proposed model began with a convolution layer followed by a rectified linear unit (ReLU) activation function and ended with a convolution layer. The model had 32 units of layers in the middle, and each unit included a convolutional layer followed by batch normalization and a ReLU. Each convolution layer had a kernel size of 3, padding of 1, and 64 channels (one channel for the final output).

The simulated dictionary signals plus randomly sampled noise from the noise dataset served as the input to train the model, and the output was the residuals (i.e., noise). The noise-free signals were obtained by subtracting the output of the model from the noisy scanned data. Independent-samples *t* test was used to measure the difference between the SNR of the training and testing datasets. Paired-samples *t* test was used to measure the difference in the SNR before and after denoising.

### 2.5. Pyramid CNN with Self-Attention for MRF Parametric Image Reconstruction

Figure 2c displays the deep learning model, which was based on a 1D CNN with a pyramidal structure. The dashed line extending from the green box indicates the detailed structure inside each green box. The input for the pyramid model was a 1D signal, and the outputs were T1 and T2* values. The backbone consisted of three convolutional layers with kernel sizes of 17, 11, and 7, and the number of channels was 128, 256, and 512, respectively. Each convolutional layer was followed by a ReLU activation function and then a dropout layer with 0.2 probability as a convolution block.

The output of T1 and T2* relaxation times had two paths. A multihead self-attention layer [20] with eight heads was first connected after each convolution block of each pathway. The expressions of the multihead self-attention are as follows:

(2) $$\mathrm{MultiHead}\left(Q,K,V\right)=\mathrm{Concat}\left({\mathrm{head}}_{1},\;\ldots ,{\;\mathrm{head}}_{h}\right)W^{O},$$

(3) $${\mathrm{head}}_{i}=\mathrm{Attention}\left(Q_{i},K_{i},V_{i}\right)=\mathrm{softmax}\left(\frac{Q_{i}K_{i}{}^{T}}{\sqrt{d_{s}}}\right)V_{i},.$$

(4) $$\text{and }\{\begin{array}{c} Q_{i}=X_{i}W_{i}^{Q}\\ K_{i}=X_{i}W_{i}^{K}\\ V_{i}=X_{i}W_{i}^{V} \end{array},\text{ and }X=X_{1},\;\ldots ,\;X_{h}$$

Equations (2)–(4) comprise the scaled dot-product self-attention with multihead. *Q*, *K*, and *V* are the query, key, and value matrices. The corresponding matrices are, $X\in {\mathbb{R}}^{l\times d_{ch}(l\times h\times d_{s})}$, $X_{i}\in {\mathbb{R}}^{l\times d_{s}}$, $W_{i}^{Q}\in {\mathbb{R}}^{d_{s}\times d_{s}}$, $W_{i}^{K}\in {\mathbb{R}}^{d_{s}\times d_{s}}$, $W_{i}^{V}\in {\mathbb{R}}^{d_{s}\times d_{s}}$, and $\;W^{O}\in {\mathbb{R}}^{hd_{s}\times d_{ch}}$, where l is the length of the signal after each convolution block; $d_{ch}$ and h are the input channels and number of heads, respectively; $d_{s}$ is $d_{ch}$ divided by h; $d_{ch}$ is 128, 256, and 512 for each convolution block; and h is 8 in our implementation.

The output of the attention layer was weighted by a learnable parameter gamma and added back to its input as the input to the next layer [26]. The next layer was a flatten layer for connecting a fully connected layer with 128 output features, followed by a ReLU, and then a fully connected layer with three output features. The final output layer was a fully connected layer with one output feature, and its input was the sum of the outputs from the different scales after being weighted by the learnable parameter gamma. The output after the learnable parameter is given by:

(5) $$Y=\text{γ}X^{a}+X$$

(6) $$\mathrm{and}\;Y={\sum^{\;}}_{i=1}^{m}\gamma_{i}X_{i}.$$

Y in Equation (5) is the input to the flatten layer, whereas $X^{a}$ is the output after the attention layer. Y in Equation (6) is the input for the final fully connected layer. Because three convolutional layers created separate scales, *m* was equal to three.

The proposed model was named the weighted pyramid dual-path CNN with attention (WPDaCNN). Three other models were employed as comparisons for the proposed model. The first was a model without the weighted parameter gamma and the self-attention layer, denoted by PDCNN. The second was a model based on PDCNN but without the pyramid structure, denoted by DCNN (only the output of the third convolutional layer was considered). The final one was a model based on DCNN but with only a single path, denoted by SCNN (the output feature for the final layer of the single path became two).

### 2.6. Experimental Setup and Two-Stage CNN Framework

Figure 2d was the flowchart of the successive process of our model. The MRF signals with noise were first inputted to the stage I model to predict the noise. The denoised MRF signals were obtained by subtracting the predictive noise from the MRF signals with noise. Then, the denoised signals were inputted to the stage II model for outputting the T1 and T2* values.

The experiment was performed on a computer with an Intel Xeon W-2102 CPU and an NVIDIA Quadro P6000 24 gigabyte GPU. The deep learning models were built based on the PyTorch package (version 1.7.1+cu110) using Python 3.8.5, and the data preprocessing for dictionary generation and matching was performed by programming platform MATLAB R2020a (MathWorks; Natick, MA, USA). Statistical analysis was performed using SPSS Statistics 24 (IBM; Armonk, NY, USA).

The L_2_ loss multiplied by 10,000 was applied to train the first stage DnCNN models. For the second-stage pyramid models, the L_1_ loss and mean absolute percentage error (MAPE) were employed and added for training. The loss functions are as follows:

(7) $${\mathrm{Loss}}_{\mathrm{stageI}}=10000\times \frac{\sum_{i=1}^{N}{\left(y_{i}{}_{residual}-y_{i}^{p}{}_{residual}\right)}^{2}}{N},$$

(8) $${\mathrm{Loss}}_{\mathrm{stageII}}=\frac{\sum_{i=1}^{N}\left|y_{i}-y_{i}^{p}\right|}{N}+\frac{100\times \sum_{i=1}^{N}\left|y_{i}-y_{i}^{p}\right|\;/y_{i}}{N}.$$

Equation (7) is the loss function for the first stage model, and Equation (8) is that for the second stage. We referred to the denoising study using the L_2_ loss for training the first stage [17], and the constant 10,000 was set empirically. The L_1_ loss for training the second stage was referenced to the literature that used the same MRF protocol as this study [13], and the MAPE term was used to balance the T1 and T2* for model learning. N is the total number of values, $y_{i}{}_{residual}$ is the true residual, $y_{i}^{p}{}_{residual}$ is the predicted residual, $y_{i}$ is the T1 and T2* values within the simulated dictionary, and $y_{i}^{p}$ is their predicted values. The value of the loss function corresponding to each stage was used as the error to identify the model with the lowest error.

**Figure 3:**
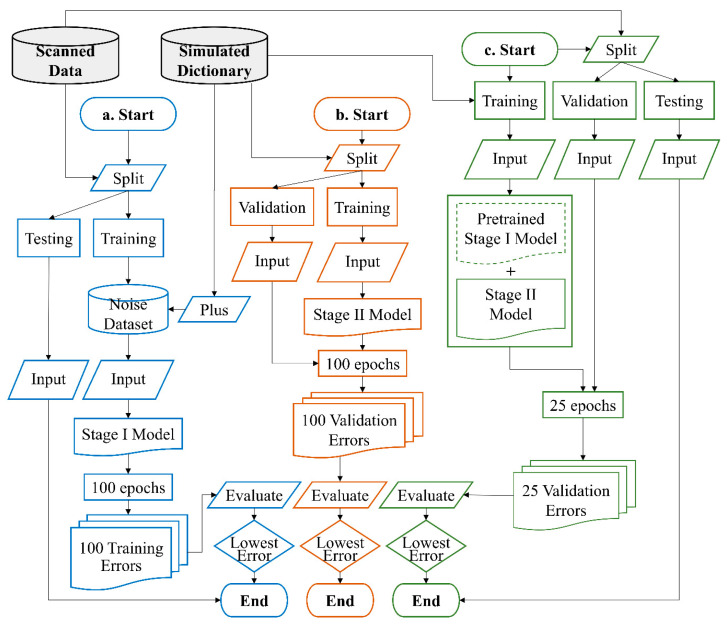
Schematic flowchart for our experiments. (**a**) Workflow for training the denoising models. (**b**) Workflow for training the pyramid models for comparison. (**c**) Workflow for training the final two-stage models. The dashed line for the pretrained stage I model indicates that the model weights were frozen and did not change during the training process.

Figure 3 presents a flowchart of our experiments. Figure 3a represents the workflow for training the DnCNN. There were 60 slices with their own unique simulated dictionaries, and thus a total of 60 models need to be trained. Because of the lengthy training time, the scanned data were split into single training and testing datasets for the experiment rather than split into multiple folds. The training and testing datasets consisted of 21 and 11 subjects from the scanned data, respectively. The noise dataset was obtained from the training dataset, as described in the Section 2.4. The DnCNN was trained for 100 epochs by inputting simulated signals plus randomly sampled values in the noise dataset. After 100 epochs, the trained model that corresponded to the lowest training error was selected as optimal. The testing dataset was then inputted to the optimal model for prediction.

Figure 3b indicates the workflow for training the models with different structures, namely, WPDaCNN, PDCNN, DCNN, and SCNN. The simulated dictionary was split into training and validation datasets according to the division described in the Section 2.3. Subsequently, each model was trained for 100 epochs, and the one with the lowest error for the validation dataset was selected as the optimal model.

Figure 3c displays the workflow for training the final two-stage model. We first connected the pretrained stage I model with the untrained stage II model and then froze the weights of the stage I model. The input for training was the simulated signal from the dictionary and did not pass through the denoising model while training. The scanned data was split into half for the validation dataset and another half for the testing dataset. After each epoch, the validation dataset was fed into the entire two-stage model for evaluating the error (loss) of the two-stage model. We observed that the second-stage model converged rapidly, and excessive training epochs led to overfitting; thus, only 25 epochs were set, and the optimal model was the one with the lowest validation error within 25 epochs. The testing dataset was then inputted to the optimal model for prediction.

Each whole-brain scan included 60 slices, with each slice corresponding to a distinct simulated dictionary. Hence, we trained a two-stage model for each slice with 598,842 entries as the input, and 60 models were eventually produced (Figure 3a,c). Because of the identical design concept of the pulse sequence for each slice, when we trained different models for comparison (i.e., WPDaCNN, PDCNN, DCNN, and SCNN), only one model of each type was trained by using a dictionary of the first slice (Figure 3b). During model training, the batch size was 500, and the optimizer employed was Adam with a learning rate of 0.01 and a scheduler with a 5% learning rate reduction per epoch. The intraclass correlation coefficient (ICC) was used to assess the consistency between the dictionary matching and prediction of the final two-stage models. The correlation coefficient was applied to test the mean and difference between the standards and predictions.

## 3. Results

### 3.1. SNR of Scan Data and after Denoising by DnCNN

**Figure 4:**
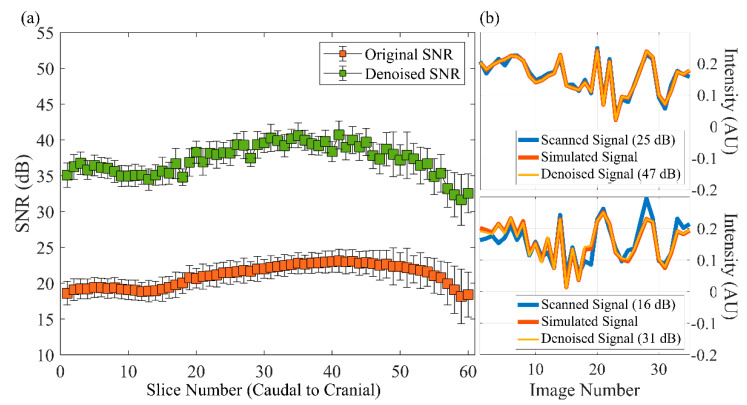
Signal-to-noise ratio (SNR) before and after denoising. (**a**) Plot of the mean (solid color box) and standard deviation (thin line bar) of the SNR in slices of the whole brain. (**b**) Two examples of the signal before and after denoising were gathered from one pixel in slice 35 (top) and slice 13 (bottom).

Figure 4a displays the corresponding SNR before and after denoising. The SNR varied from slice to slice, with lower SNRs in the cranial and caudal portions and higher SNRs in the middle. Figure 4b contains two examples of the scanned signal after denoising. The noise was effectively removed after denoising, and the SNR increased (25 dB vs. 47 dB and 16 dB vs. 31 dB). Table 1 presents the SNR and statistics before and after the denoising by the DnCNN for the training and testing datasets. The results for various tissue types were obtained after applying the tissue masks that were created by the automatic and manual segmentation mentioned in the Section 2.3. The SNRs in both the training and testing datasets increased, and the increases after denoising were statistically significant (*p* < 0.001). The SNRs of GM and WM were similar, whereas the SNR of CSF was lower than that of GM and WM. Regarding the differences in mean SNR between the training and testing datasets, the *p* values were 0.40 and 0.32 for the original and denoised SNRs, respectively. This result suggested that the mean SNRs of the training and testing datasets were not significantly different, either before or after denoising. Therefore, the model performed well in the testing dataset.

### 3.2. Performance of the Pyramid CNN Models

**Figure 5:**
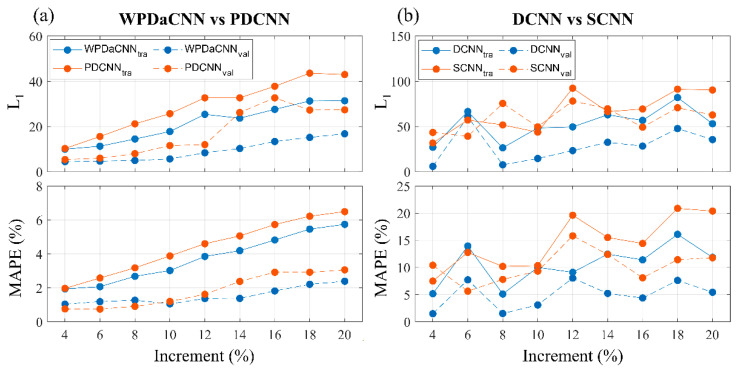
Performance of pyramid models trained under different dictionary increments. The solid line is the model prediction by training data, and the dashed line is that of the testing data. (**a**) L_1_ and MAPE of the WPDaCNN and PDCNN models. (**b**) L_1_ and MAPE of the DCNN and SCNN models.

For the first division, models learned well on the training dataset but poorly on the validation dataset. The mean MAPE of all models on the training dataset was 1.4%, and that on the validation dataset was 54.8%. For the second division, Figure 5 presents the pyramid model performance for different dictionary increments. As the increment increased, the losses of WPDaCNN and PDCNN increased smoothly, but the losses of DCNN and SCNN increased ruggedly. For the L_1_ loss, WPDaCNN was the model with the optimal performance under all dictionary increments. The lowest L_1_ loss was 10 ms and 4.5 ms for the training and validation, respectively, at the dictionary with the densest increment. Compared with SCNN, DCNN had lower losses, except for training losses at the increments of six and eight and validation losses at six.

### 3.3. MRF Parametric Maps Reconstruction by the Two-Stage Model

The dictionary matching using the inner product by the CPU required 1.5 min to reconstruct a slice, and the previous model with the same MRF protocol as this study by the CPU required 0.08 s [13]. The time required for the GPU with a two-stage model to reconstruct a slice was 0.02 s. Table 2 and Table 3 present the statistical analysis of T1 and T2* values from 32 subjects by standard dictionary matching and that by the proposed two-stage model. Results for various tissue types were obtained by applying the corresponding tissue mask derived from the automatic and manual segmentation. In both validation and testing datasets, all ICCs were higher than 0.94 in T1 and T2* relaxation times for all tissues. The MAPE decreased by approximately a factor of two after denoising for all tissue types. In GM, WM, and MS lesions, the MAPE was less than 3.2% for T1 and 2.8% for T2* with denoising. CSF had a much higher MAPE compared with other tissue types. The overall MAPE with the denoising for the whole brain was approximately 6% and 4% for the T1 and T2* values, respectively. Most of the overall increase in error was contributed by CSF. The previous model based on U-Net and scan data for training was MAPE of five to ten [13].

**Figure 6:**
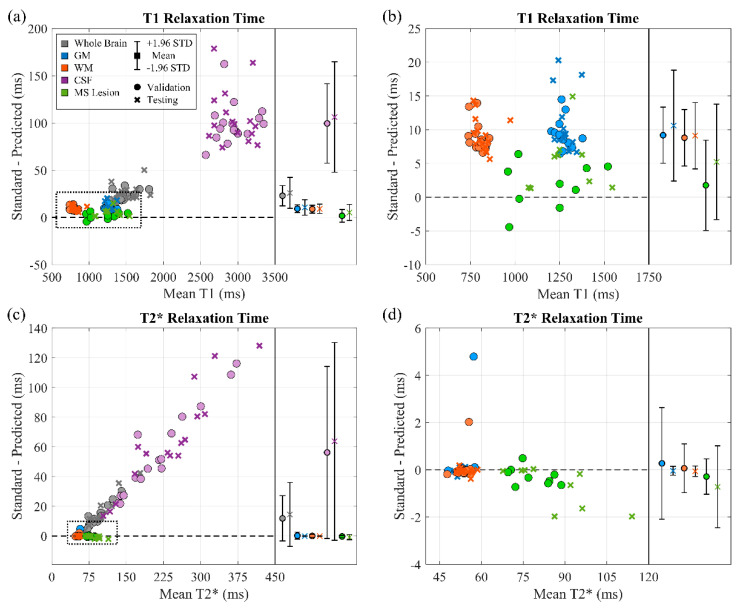
Bland-Altman plot for mean T1 and T2* relaxation times of 32 subjects (18 with MS lesions) for the whole brain and different tissues. (**a**,**c**) Plot for all tissues. (**b**,**d**) Cropped views of GM, WM, and MS lesion shown in (**a**) and (**c**).

**Figure 7:**
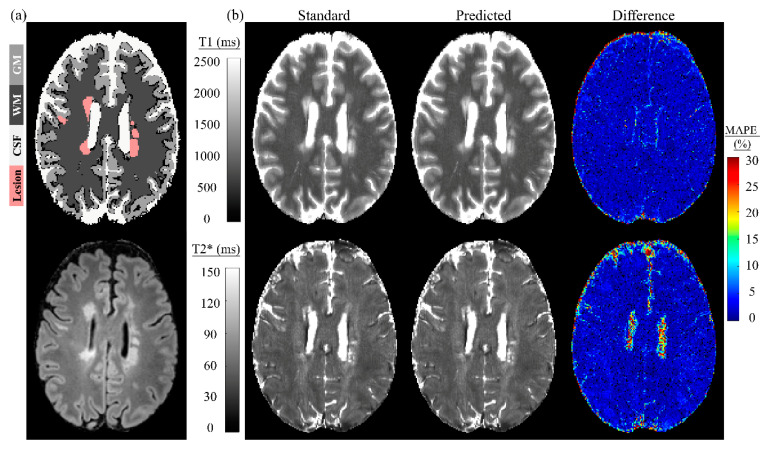
Magnetic resonance fingerprinting parametric maps of a single slice in an MS patient matched by the simulated dictionary (standard) and predicted by the proposed model. (**a**) Top is the tissue masks; bottom is the FLAIR. (**b**) Standard maps by dictionary matching, predicted maps by the proposed two-stage model, and difference maps between them.

Figure 6 displays a Bland–Altman plot for all subjects of dictionary matching and the two-stage model. The fifth and sixth columns of Table 2 and Table 3 lists the mean and difference (with standard deviations) between them. A significant positive correlation was observed for the whole brain for T1 and T2* in the validation dataset and T2* in the testing dataset. The significant positive correlation also appeared in the CSF for T2* for both validation and testing datasets. A significant negative correlation was observed for the MS lesion for T2* in the testing dataset. In both validation and testing datasets, the mean difference was less than or equal to 10 ms for GM and WM, and 5 ms for the MS lesion, for T1. The mean difference was less than or equal to 0.7 ms for GM, WM, and the MS lesion for T2* in both validation and testing datasets. Figure 7 depicts a single slice from an MS patient for the tissue masks, FLAIR, standard and predicted maps for T1 and T2*, and their corresponding difference maps. The standard maps were obtained by dictionary matching using the inner-product method, and the predicted maps were gathered by the proposed two-stage model. The MAPE for GM, WM, and MS lesions was low. The MAPE was higher for the CSF region compared with other tissue types in the difference map, especially for T2*.

## 4. Discussion

Herein, we propose a two-stage model for predicting parametric maps of MRF-EPI. The prediction results achieved a MAPE of equal to or less than 3% from the standard dictionary matching for GM, WM, and MS lesions. In our approach, the first stage used MRF signal denoising, and the second stage used regression of the simulated signal by the Bloch equation [18]. The model’s prediction error with denoising was approximately one-half that without denoising, and in this, we demonstrated the importance of removing the noise. Furthermore, the pyramid model with self-attention learned well on the simulated signal and achieved MAPE of approximately 2% and 1% for the training and validation datasets, respectively, for the dictionary with the densest increment. Our proposed model accurately reconstructed parametric maps of MRF-EPI and can therefore replace the computationally expensive inner-product dictionary matching method.

Noise is an unavoidable problem when MRI is conducted using fast imaging techniques, and acquisition speed and SNR are perennial tradeoffs. Several approaches have been proposed for MRI denoising [27]. In general, denoising techniques are based on specific assumptions to model prior properties, such as inherent pattern redundancy and sparsity. The disadvantages of such modeling are that obtaining high performance is computationally expensive and that several manual parameters must be selected [17]. Unlike prior-based approaches, deep learning–based DnCNN is both effective and time efficient. A previous study demonstrated that by filtering the MRF baseline images, the image quality improved for parametric maps [24]. We also performed noise reduction on the MRF baseline image, but we did so on the signal evolution of each pixel instead of on 2D images. SNRs for both the training and testing datasets increased by nearly twice the original SNRs after denoising. No significant difference was observed between the training and testing datasets before and after denoising in our experiments. This result demonstrated that the DnCNN performed well in handling MRF signals with noise for both training and testing datasets. In addition, we observed a decrease in the SNR on the cranial and caudal sides, which conforms with observations in previous studies [28,29].

In learning simulated signals with different increments, the error in model prediction on both training and validation datasets increased as the increment increased. We observed that the PDCNN and WPDaCNN had fewer errors and a smoother error trend than did the DCNN and SCNN. From this result, we observed that the model with the pyramid structure was more stable than the model without the pyramid structure. In addition, in the first division type of our experiments, the model was made to learn certain T1 and T2* ranges of simulated signals and to predict the data outside the simulated scope as validation. This approach resulted in poor prediction for the validation dataset. This result demonstrated that the model did not learn the Bloch equation simulation [18] well. The model must be made to learn all the expected ranges for the simulated signals to ensure accurate predictions. Moreover, regarding the performance of the PDCNN and WPDaCNN, the validation loss was lower than the training loss at any increment, indicating that the model accurately regressed the learned data within the range of T1 and T2* contained in the training dictionary. That is, once the model learned the dictionary with a 4% increment, it was able to regress the T1 and T2* of the dictionary with a 2% increment well. Deep learning models perform better with a single output compared with multiple outputs [13]. In our experimental results, the overall performance of the DCNN was superior to that of the SCNN, which demonstrated that the dual-path for outputting T1 and T2* was beneficial in improving the model performance.

To address the problem of noise, previous studies have used Gaussian noise to test the performance of their model [8,24], but actual MRI noise distributions are non-Gaussian [30,31]. Thus, we created a noise dataset on the basis of the difference between simulated and scanned data and randomly sampled the data from this dataset to train our deep learning model. Furthermore, models trained by L_1_ loss were reported to perform more favorably in MRF image reconstruction compared with other loss functions [13]. Hence, we concurrently used L_1_ loss and MAPE loss to avoid the model’s overfitting to either T1 or T2* values (Equation (8)). In addition, studies have demonstrated the power of CNNs and the ability of RNNs to outperform CNNs in MRF image reconstruction [9,10]. For natural language processing, Transformer, which relies entirely on the self-attention mechanism, has been proposed as having a lower computational cost and more advanced performance than RNNs [20]. We combined a CNN with the self-attention mechanism to make the model learn the correlations among features captured by the CNN. Furthermore, the performance of a two-stage model is superior to that of a single-stage one for object detection but at the expense of computational speed [32]. Our results also indicated that the two-stage model with noise reduction outperformed the one-stage model without noise reduction. Moreover, the computation time of the model in the GPU (0.02 s) for predicting a single slice was 4500-fold faster than that of the commonly used inner-product matching in the CPU (90 s). Finally, although correlations were observed in the Bland–Altman analysis, the MAPE for clinically interesting tissues (GM, WM, and MS lesions) was less than or equal to 3%, and the mean T1 and T2* values of these tissues are consistent with those in previous studies [1,33,34,35,36].

Clinical MRI relies on qualitative imaging, which can require one hour to obtain multiple contrast weightings. Prolonged scanning is a burden for patients who cannot recline for long periods and may record motion artifacts because of patient movement. Additionally, qualitative imaging can be affected by the scanner and imaging parameters used, which hinders disease follow-up. By contrast, MRF quantitative imaging can generate multiple relaxation time maps in only a few minutes of scanning time. MRF has been demonstrated to have high repeatability and reproducibility [37,38]. MRF is a favorable approach to obtaining quantitative MR relaxation measurements. In addition, quantitative MR relaxometry can synthesize conventional contrast weightings [2,3], which can be useful for adherence to current clinical diagnostic standards. Furthermore, quantitative MRI relaxometry–based tissue segmentation was reported to have favorable repeatability [39] and can be beneficial in clinical settings for tracking the time course of a disease. With improvements addressing the drawback of the long reconstruction time of MRF, this approach is expected to replace the conventional weighted imaging currently used in clinical practice. In this study, we propose a two-stage model that is able to learn the simulated dictionary with dense increment and more quickly than dictionary matching. Our model can accelerate MRF reconstruction and thus increase the feasibility of MRF for clinical applications.

This study has some limitations. First, the gold standard we applied to evaluate the accuracy of our models was the use of the parametric maps by dictionary matching, and no other reference quantitative method was used. However, the quantification accuracy of MRF-EPI by dictionary matching was validated with a phantom and had good agreement [22,23]. Second, the prediction time we reported in the Results section was for only one slice, and approximately 30 s were required to compute 60 slices consecutively. This result was due to the continuous GPU computing also involving memory usage and data transfer time. Finally, because of the design of MRF-EPI, the simulated dictionary differed by slice. Therefore, we trained a total of 60 models corresponding to each slice, and this required training time and space to store the trained weights for the model. Approximately nine days were required to train the denoising model for stage I, and 18 h to train different pyramid models for comparison. Regarding the final two-stage model, an excessive epoch number led to poor model prediction for the scanned data because of overfitting. Therefore, we used a relatively small number of training sessions (25 epochs), and approximately two days were required for model training. Regarding storage space, space requirements were smaller compared with those for the simulated dictionary (15 megabytes for the model weights and 203 megabytes for the dictionary of each slice).

In this study, we proposed a two-stage model. The MRF signal noise reduction was for the first stage, and the T1 and T2* value prediction was for the second stage. The results showed that noise removal was very beneficial for predicting the T1 and T2* values. Compared with other studies, we used real noise and the simulation dictionary to train the model to ensure generalizability. Our proposed model was designed using a 1D architecture, which required model training for each slice. If the model is designed in 3D, a single model will be able to cover the whole brain. However, compared with the multi-model approach, the single model has fewer parameters for learning, and it is conceivable that the noise reduction performance may be worse. We used the denoising CNN proposed by Zhang et al. in 2017 [17]. Other advanced denoising deep learning models, such as a denoising autoencoder [40], are available and can be used in MRF studies in the future to improve the model performance in noise reduction. Besides, MRF using EPI fast imaging is sensitive to magnetic field inhomogeneity and can have distortion artifacts at the air-tissue interface. A common approach for distortion correction is image registration [41]. In addition, MRI image analysis often requires the segmentation of tissues such as GM, WM, CSF, and lesion to observe the correlated volumetric changes [42]. Deep learning is well established in image registration and segmentation, such as VoxelMorph [43], which used the spatial transformer function, and U-Net [44], a well-known architecture commonly used for medical image segmentation. In the future, a multi-task deep learning model for MRF can be added to specifically handle the image denoising, registration, and segmentation tasks to achieve a one-stop efficient MRF image reconstruction and enhance the value of MRF in clinical applications.

## 5. Conclusions

In conclusion, we effectively removed the noise from MRF-EPI in a 1D manner and thus improved the performance of a deep learning model in the regression task for MRF parametric map reconstruction. The proposed model achieved a prediction error equal to or less than 3% in the T1 and T2* map for tissues of clinical interest, such as GM, WM, and MS lesions. Compared with the 1.5 min required for the CPU computation using the inner-product method, the proposed model can achieve a computation speed of 0.02 s for a slice in the GPU. Our proposed two-stage model, trained with dense-increment simulated dictionaries, can accelerate image reconstruction and reduce the space required by dictionaries, thus improving imaging efficiency. Future research can target deep learning models that incorporate image processing, such as image registration and segmentation, to overcome the distortion and measure the brain volumetry for facilitating MRF in clinical applications.

## Figures and Tables

**Table 1:** SNR before and after the denoising model.

	Training			Testing		
Tissue Type	Original SNR	Denoised SNR	*p* Value	Original SNR	Denoised SNR	*p* Value
Whole Brain	21.33 ± 1.45	37.75 ± 1.55	<0.001 *	21.78 ± 1.41	38.31 ± 1.34	<0.001 *
GM	22.22 ± 1.51	39.10 ± 1.56	<0.001 *	23.01 ± 1.52	39.86 ± 1.31	<0.001 *
WM	22.44 ± 1.49	40.03 ± 1.68	<0.001 *	22.79 ± 1.69	40.48 ± 1.60	<0.001 *
CSF	18.97 ± 1.60	33.29 ± 1.59	<0.001 *	18.75 ± 1.30	33.31 ± 1.34	<0.001 *

GM = gray matter; WM = white matter; CSF = cerebrospinal fluid; MS = multiple sclerosis; SNR = signal-to-noise ratio. The unit of SNR is in dB. “*” indicates that the *p* value is less than 0.05.

**Table 2:** Statistical analysis of T1 relaxation times between dictionary matching and the proposed two-stage model for each tissue of scanned data.

**Validation (7 Healthy and 9 with MS)**								
	**Standard (ms)**	**Predicted (ms)**	**ICC**	**MAPE (%)**	**Mean (ms)**	**Difference (ms)**	**R**	***p* Value**
WB	1483 ± 147	1461 ± 146	1.00	5.9 (12.6)	1472 ± 145	22.9 ± 5.5	0.53	<0.05 *
GM	1278 ± 42	1269 ± 42	1.00	3.0 (7.1)	1273 ± 42	9.2 ± 2.1	−0.22	0.41
WM	803 ± 35	794 ± 36	1.00	3.0 (6.8)	799 ± 36	8.8 ± 2.1	−0.45	0.08
CSF	2993 ± 233	2894 ± 229	1.00	7.4 (15.1)	2943 ± 231	99.5 ± 21.5	0.17	0.54
MSL	1194 ± 208	1192 ± 207	1.00	1.7 (3.9)	1193 ± 207	1.8 ± 3.4	0.28	0.46
**Testing (7 Healthy and 9 with MS**)								
WB	1521 ± 153	1496 ± 151	1.00	6.2 (12.9)	1509 ± 152	25.9 ± 8.4	0.27	0.31
GM	1286 ± 42	1276 ± 43	1.00	3.1 (7.2)	1281 ± 42	10.6 ± 4.2	−0.16	0.57
WM	825 ± 51	816 ± 51	1.00	3.2 (7.0)	820 ± 51	9.1 ± 2.5	−0.25	0.35
CSF	3003 ± 224	2897 ± 230	1.00	7.4 (14.5)	2950 ± 226	106.3 ± 29.8	−0.22	0.41
MSL	1284 ± 152	1279 ± 151	1.00	1.9 (4.2)	1282 ± 152	5.2 ± 4.4	0.10	0.81

WB = whole brain; GM = gray matter; WM = white matter; CSF = cerebrospinal fluid; MSL = lesion of multiple sclerosis; ICC = intraclass correlation coefficient; MAPE = mean absolute percentage error. “*” indicates that the *p* value is less than 0.05. The values in parentheses in the MAPE column are the results without noise removal. The difference is from the pairwise pixel-value difference.

**Table 3:** Statistical analysis of T2* relaxation times between dictionary matching and the proposed two-stage model for each tissue of scanned data.

**Validation (7 Healthy and 9 with MS)**								
	**Standard (ms)**	**Predicted (ms)**	**ICC**	**MAPE (%)**	**Mean (ms)**	**Difference (ms)**	**R**	***p* Value**
WB	95 ± 26	83 ± 18	0.97	4.2 (11.3)	89 ± 22	11.9 ± 7.8	0.96	<0.001 *
GM	53 ± 3	53 ± 2	0.94	2.6 (6.0)	53 ± 2	0.3 ± 1.2	0.49	0.05
WM	53 ± 2	53 ± 2	0.98	2.2 (5.8)	53 ± 2	0.1 ± 0.5	0.35	0.18
CSF	245 ± 90	189 ± 61	0.96	9.3 (20.1)	217 ± 75	56.3 ± 29.5	0.96	<0.001 *
MSL	78 ± 7	79 ± 7	1.00	2.0 (4.8)	79 ± 7	−0.3 ± 0.4	−0.43	0.25
**Testing (7 Healthy and 9 with MS)**								
WB	104 ± 35	89 ± 24	0.97	4.6 (10.5)	96 ± 30	14.5 ± 11.0	0.96	<0.001 *
GM	53 ± 2	53 ± 2	1.00	2.6 (5.6)	53 ± 2	−0.0 ± 0.1	0.40	0.12
WM	54 ± 2	54 ± 2	1.00	2.3 (5.2)	54 ± 2	−0.1 ± 0.1	−0.21	0.43
CSF	268 ± 101	204 ± 70	0.96	10.2 (18.9)	236 ± 85	63.8 ± 34.0	0.93	<0.001 *
MSL	86 ± 14	87 ± 15	1.00	2.8 (6.5)	87 ± 14	−0.7 ± 0.9	−0.71	<0.05 *

WB = whole brain; GM = gray matter; WM = white matter; CSF = cerebrospinal fluid; MSL = lesion of multiple sclerosis; ICC = intraclass correlation coefficient; MAPE = mean absolute percentage error. “*” indicates that the *p* value is less than 0.05. The values in parentheses in the MAPE column are the results without noise removal. The difference is from the pairwise pixel-value difference.

## Data Availability

The data presented in this study is not publicly available due to patient privacy concerns; publication would not be covered by the IRB statement.

## References

[1] Cheng H.L., Stikov N., Ghugre N.R., Wright G.A. (2012). Practical Medical Applications of Quantitative MR Relaxometry. J. Magn. Reson. Imaging.

[2] Feng L., Ma D., Liu F. (2020). Rapid MR Relaxometry Using Deep Learning: An Overview of Current Techniques and Emerging Trends. NMR Biomed..

[3] Ji S., Yang D., Lee J., Choi S.H., Kim H., Kang K.M. (2020). Synthetic MRI: Technologies and Applications in Neuroradiology. J. Magn. Reson. Imaging.

[4] Ma D., Gulani V., Seiberlich N., Liu K., Sunshine J.L., Duerk J.L., Griswold M.A. (2013). Magnetic Resonance Fingerprinting. Nature.

[5] McGivney D.F., Boyacıoğlu R., Jiang Y., Poorman M.E., Seiberlich N., Gulani V., Keenan K.E., Griswold M.A., Ma D. (2020). Magnetic Resonance Fingerprinting Review Part 2: Technique and Directions. J. Magn. Reson. Imaging.

[6] McGivney D.F., Pierre E., Ma D., Jiang Y., Saybasili H., Gulani V., Griswold M.A. (2014). SVD Compression for Magnetic Resonance Fingerprinting in the Time Domain. IEEE Trans Med. Imaging.

[7] Yang M., Ma D., Jiang Y., Hamilton J., Seiberlich N., Griswold M.A., McGivney D. (2018). Low Rank Approximation Methods for MR Fingerprinting with Large Scale Dictionaries. Magn. Reson. Med..

[8] Cohen O., Zhu B., Rosen M.S. (2018). MR Fingerprinting Deep RecOnstruction NEtwork (DRONE). Magn. Reson. Med..

[9] Hoppe E., Thamm F., Korzdorfer G., Syben C., Schirrmacher F., Nittka M., Pfeuffer J., Meyer H., Maier A. (2019). Magnetic Resonance Fingerprinting Reconstruction Using Recurrent Neural Networks. Stud. Health Technol..

[10] Hoppe E., Körzdörfer G., Würfl T., Wetzl J., Lugauer F., Pfeuffer J., Maier A. (2017). Deep Learning for Magnetic Resonance Fingerprinting: A New Approach for Predicting Quantitative Parameter Values from Time Series. Ger. Med. Data Sci. Vis. Bridges.

[11] Balsiger F., Jungo A., Scheidegger O., Carlier P.G., Reyes M., Marty B. (2020). Spatially Regularized Parametric Map Reconstruction for Fast Magnetic Resonance Fingerprinting. Med. Image Anal..

[12] Fang Z., Chen Y., Liu M., Xiang L., Zhang Q., Wang Q., Lin W., Shen D. (2019). Deep Learning for Fast and Spatially Constrained Tissue Quantification From Highly Accelerated Data in Magnetic Resonance Fingerprinting. IEEE Trans. Med. Imaging.

[13] Hermann I., Martinez-Heras E., Rieger B., Schmidt R., Golla A.K., Hong J.S., Lee W.K., Yu-Te W., Nagtegaal M., Solana E. (2021). Accelerated White Matter Lesion Analysis Based on Simultaneous T1 and T2* Quantification Using Magnetic Resonance Fingerprinting and Deep Learning. Magn. Reson. Med..

[14] Yang M., Jiang Y., Ma D., Mehta B.B., Griswold M.A. (2020). Game of Learning Bloch Equation Simulations for MR Fingerprinting. arXiv.

[15] Chen D., Golbabaee M., Gomez P.A., Menzel M.I., Davies M.E. (2019). A Fully Convolutional Network for MR Fingerprinting. arXiv.

[16] Li G., Zrimec J., Ji B., Geng J., Larsbrink J., Zelezniak A., Nielsen J., Engqvist M.K. (2021). Performance of Regression Models as a Function of Experiment Noise. Bioinform. Biol. Insights.

[17] Zhang K., Zuo W., Chen Y., Meng D., Zhang L. (2017). Beyond a Gaussian Denoiser: Residual Learning of Deep CNN for Image Denoising. IEEE Trans Image Process.

[18] Bloch F. (1946). Nuclear Induction. Phys. Rev..

[19] Lin T.-Y., Dollár P., Girshick R., He K., Hariharan B., Belongie S. Feature Pyramid Networks for Object Detection. Proceedings of the IEEE Computer Society.

[20] Vaswani A., Shazeer N., Parmar N., Uszkoreit J., Jones L., Gomez A.N., Kaiser Ł., Polosukhin I. (2017). Attention Is All You Need. Proceedings of the 31st International Conference on Neural Information Processing Systems.

[21] Wang X., Girshick R., Gupta A., He K. Non-Local Neural Networks. Proceedings of the 2018 IEEE/CVF Conference on Computer Vision and Pattern Recognition.

[22] Rieger B., Zimmer F., Zapp J., Weingartner S., Schad L.R. (2017). Magnetic Resonance Fingerprinting Using Echo-Planar Imaging: Joint Quantification of T1 and T2* Relaxation Times. Magn. Reson. Med..

[23] Rieger B., Akcakaya M., Pariente J.C., Llufriu S., Martinez-Heras E., Weingartner S., Schad L.R. (2018). Time Efficient Whole-Brain Coverage with MR Fingerprinting Using Slice-Interleaved Echo-Planar-Imaging. Sci. Rep..

[24] Hermann I., Chacon-Caldera J., Brumer I., Rieger B., Weingartner S., Schad L.R., Zollner F.G. (2020). Magnetic Resonance Fingerprinting for Simultaneous Renal T1 and T2* Mapping in a Single Breath-Hold. Magn. Reson. Med..

[25] Ashburner J., Barnes G., Chen C.-C., Daunizeau J., Flandin G., Friston K., Kiebel S., Kilner J., Litvak V., Moran R. (2014). SPM12 Manual.

[26] Zhang H., Goodfellow I., Metaxas D., Odena A. (2019). Self-Attention Generative Adversarial Networks. arXiv.

[27] Mohan J., Krishnaveni V., Guo Y. (2014). A Survey on the Magnetic Resonance Image Denoising Methods. Biomed. Signal Process. Control.

[28] Larsson E.-M., Nilsson H., Holtås S., Ståhlberg F. (1989). Coil Selection for Magnetic Resonance Imaging of the Cervical and Thoracic Spine Using a Vertical Magnetic Field. Acta Radiol..

[29] Reiss-Zimmermann M., Gutberlet M., Köstler H., Fritzsch D., Hoffmann K.-T. (2013). Improvement of SNR and Acquisition Acceleration Using a 32-Channel Head Coil Compared to a 12-Channel Head Coil at 3T. Acta Radiol..

[30] Gudbjartsson H., Patz S. (1995). The Rician Distribution of Noisy MRI Data. Magn. Reson. Med..

[31] Nowak R.D. (1999). Wavelet-Based Rician Noise Removal for Magnetic Resonance Imaging. IEEE Trans. Image Processing.

[32] Soviany P., Ionescu R.T. Optimizing the Trade-Off between Single-Stage and Two-Stage Deep Object Detectors Using Image Difficulty Prediction. Proceedings of the 2018 20th International Symposium on Symbolic and Numeric Algorithms for Scientific Computing (SYNASC).

[33] Blystad I., Håkansson I., Tisell A., Ernerudh J., Smedby Ö., Lundberg P., Larsson E.-M. (2016). Quantitative MRI for Analysis of Active Multiple Sclerosis Lesions without Gadolinium-Based Contrast Agent. Am. J. Neuroradiol..

[34] Krüger G., Glover G.H. (2001). Physiological Noise in Oxygenation-Sensitive Magnetic Resonance Imaging. Magn. Reson. Med..

[35] Péran P., Hagberg G., Luccichenti G., Cherubini A., Brainovich V., Celsis P., Caltagirone C., Sabatini U. (2007). Voxel-Based Analysis of R2* Maps in the Healthy Human Brain. J. Magn. Reson. Imaging.

[36] Wansapura J.P., Holland S.K., Dunn R.S., Ball W.S. (1999). NMR Relaxation Times in the Human Brain at 3.0 Tesla. J. Magn. Reson. Imaging.

[37] Jiang Y., Ma D., Keenan K.E., Stupic K.F., Gulani V., Griswold M.A. (2017). Repeatability of Magnetic Resonance Fingerprinting T1 and T2 Estimates Assessed Using the ISMRM/NIST MRI System Phantom. Magn. Reson. Med..

[38] Körzdörfer G., Kirsch R., Liu K., Pfeuffer J., Hensel B., Jiang Y., Ma D., Gratz M., Bär P., Bogner W. (2019). Reproducibility and Repeatability of MR Fingerprinting Relaxometry in the Human Brain. Radiology.

[39] Andica C., Hagiwara A., Hori M., Nakazawa M., Goto M., Koshino S., Kamagata K., Kumamaru K.K., Aoki S. (2018). Automated Brain Tissue and Myelin Volumetry Based on Quantitative MR Imaging with Various In-Plane Resolutions. J. Neuroradiol..

[40] Lee W.-H., Ozger M., Challita U., Sung K.W. (2021). Noise Learning Based Denoising Autoencoder. IEEE Commun. Lett..

[41] Wang S., Peterson D.J., Gatenby J.C., Li W., Grabowski T.J., Madhyastha T.M. (2017). Evaluation of Field Map and Nonlinear Registration Methods for Correction of Susceptibility Artifacts in Diffusion MRI. Front. Neuroinformatics.

[42] Giorgio A., De Stefano N. (2013). Clinical Use of Brain Volumetry. J. Magn. Reson. Imaging.

[43] Balakrishnan G., Zhao A., Sabuncu M.R., Guttag J., Dalca A.V. (2019). VoxelMorph: A Learning Framework for Deformable Medical Image Registration. IEEE Trans. Med. Imaging.

[44] Ronneberger O., Fischer P., Brox T. (2015). U-Net: Convolutional Networks for Biomedical Image Segmentation. arXiv.

